# Traumatic Nerve Lesions Require Pre-operative and Post-operative Assessment Through Nerve Conduction Studies and Electromyography

**DOI:** 10.5704/MOJ.2407.014

**Published:** 2024-07

**Authors:** J Finsterer

**Affiliations:** Neurological Department, Neurology and Neurophysiology Center, Vienna, Austria

Dear editor,

We read with interest the article by Gupta *et al* about 18 patients with occupational nerve injuries caused by metallic foreign bodies while operating lathes^1^. Two patients were found to have more than one nerve injury and one patient had an associated vascular injury^1^. The most commonly injured nerve was the median nerve. The most common site for nerve injury was the forearm^1^. Combined lesions most commonly affected the ulnar and median nerves^1^. It was concluded that the costs of traumatic peripheral nerve injuries are significant because they occur particularly frequent in young, previously healthy and economically active people^1^. The study is impressive, but some points require further discussion.

The first point is that neither nerve conduction studies (NCSs) nor needle electromyography (EMG) results have been reported^1^. In order to assess not only the site of injury but also the degree of injury, it is imperative to perform NCSs and EMG in patients with traumatic nerve lesions. NCSs may also be able to assess whether neurapraxia, axonotmesis, or neurotmesis is present^2^. In addition, NCSs and EMG assessment of whether re-innervation is present and whether graft implantation was beneficial or unsuccessful are required^3^.

The second point is that the outcome was not reported in detail. We should know whether “time to repair” means time to complete recovery or time to improvement with remaining deficits. According to figure 1, at least two patients were followed up for one year. It would be desirable to know the long-term outcome of all 18 patients. The long-term outcome of the two patients who underwent nerve transfer is also of particular interest. We should know not only the level of muscle strength in the muscles supplied by the affected nerve but also whether there were muscle wasting or sensory deficits in the supplied areas.

Third, there are differences in the number of patients in Table I^1^. According to the methods section, 18 patients were included, but in Table I the number of patients for the item “nerve involved” is 20. This discrepancy should be resolved. Only 16 patients are listed for the item “time to repair”^1^. What were the outcomes of the two remaining patients? Were long-term outcome results available for more than only two patients?

A fourth point concerns the conclusions. In order to prevent the nerve injuries described, it is not only imperative to train lathe workers, but also to force them to wear protective suits or to better automate the production process and install robots.

In summary, the excellent study has limitations that should be addressed before drawing final conclusions. Clarifying the weaknesses would strengthen the conclusions and could improve the study. To evaluate traumatic nerve injuries, patients should undergo NCSs and EMG prior to surgical repair to locate the site of injury and grade the severity of the lesion. NCS and EMG may also be useful to assess the progression and degree of re-innervation during follow-up.
